# Perceived Health Benefits and Willingness to Pay for Parks by Park Users: Quantitative and Qualitative Research

**DOI:** 10.3390/ijerph14050529

**Published:** 2017-05-15

**Authors:** Claire Henderson-Wilson, Kah-Ling Sia, Jenny Veitch, Petra K Staiger, Penny Davidson, Peter Nicholls

**Affiliations:** 1 Deakin University, Geelong Australia, Health Nature and Sustainability Research Group, School of Health and Social Development, Deakin University, 221 Burwood Highway, Burwood, VIC 3125, Australia; 2 Deakin University, Geelong Australia, Deakin Health Economics, School of Health and Social Development, Deakin University, 221 Burwood Highway, Burwood, VIC 3125, Australia; Kah-Ling.Sia@dhhs.vic.gov.au; 3 Deakin University, Geelong Australia, Institute for Physical Activity and Nutrition (IPAN), Deakin University, 221 Burwood Highway, Burwood, VIC 3125, Australia; jenny.veitch@deakin.edu.au; 4 Deakin University, Geelong Australia, School of Psychology, Deakin University, 221 Burwood Highway, Burwood, VIC 3125, Australia; petra.staiger@deakin.edu.au; 5 Parks and Leisure Australia, PLA National Office, 207 The Parade, Norwood, SA 5067, Australia; pla.advisory@parksleisure.com.au; 6 Parks and Leisure Australia, P.O. Box 536, Walkerville, SA 5081, Australia; peter@australiaspeoplegardener.com.au

**Keywords:** parks, health, wellbeing, economic value

## Abstract

Whilst a growing body of evidence demonstrates people derive a range of health and wellbeing benefits from visiting parks, only a limited number of attempts have been made to provide a complementary economic assessment of parks. The aim of this exploratory study was to directly estimate the perceived health and wellbeing benefits attained from parks and the economic value assigned to parks by park users in Victoria, Australia. The research employed a mixed methods approach (survey and interviews) to collect primary data from a selection of 140 park users: 100 from two metropolitan parks in Melbourne and 40 from a park on the urban fringe of Melbourne, Victoria. Our findings suggest that park users derive a range of perceived physical, mental/spiritual, and social health benefits, but park use was predominantly associated with physical health benefits. Overall, our exploratory study findings suggest that park users are willing to pay for parks, as they highly value them as places for exercising, socialising, and relaxing. Importantly, most people would miss parks if they did not exist. The findings aim to provide park managers, public health advocates, and urban policy makers with evidence about the perceived health and wellbeing benefits of park usage and the economic value park visitors place on parks.

## 1. Introduction

Increasingly people are living in urban areas. As cities grow, land is prized at a premium and with it come challenges to develop sustainable infrastructure. Parks and green spaces are an often-deferred element in this, as the health and economic benefits of parks are largely under-rated and not well-understood and evidence is mostly local and specific. In times of increasing obesity, cardiovascular disease, and mental health disorders, we need to fully understand the benefits of parks so that we optimise the preventative and remedial impacts they have on people’s health and wellbeing.

Like many western nations, parks are freely available in most neighbourhoods in Australia thanks to the foresight of key decision makers which has resulted in quality open space. Without this foresight, our cities might look quite different. For example, without Tom Uren’s influence in Sydney, the restoration and re-use of derelict inner city areas such as the Glebe Estate and Woolloomooloo, the reclamation of Duck Creek and the creation of the Chipping Norton Lakes Scheme would not have occurred. Parks offer numerous psychological, physical, social, economic, and environmental benefits to residents of all ages [1,2,3,4] and there is growing international research on park use and its associated health and wellbeing benefits. A Danish survey found a correlation between proximal green spaces (parks) and lower levels of obesity and stress [5]. Another Chicago based survey showed “parks spaces are found to directly mitigate stress by fostering social support” [6] (p. 1201). 

There is also growing literature identifying links between availability of neighbourhood parks and enhanced physical activity [7,8,9,10] and obesity-related health indicators [11,12], and also relationships with perceived general health [13], mental health [14,15,16], morbidity [17], and mortality [18,19]. Furthermore, a study that included pooled data from 14 cities in ten high and low-middle income countries including the U.S., Belgium, Czech Republic, Denmark, Hong Kong, New Zealand, the United Kingdom, Brazil, Colombia, and Mexico found the number of parks located within a 0.5-km buffer from home was positively associated with physical activity [10]. Despite this growing evidence, as Nielsen and Hansen [5] note, further research is needed, especially Australian research, if the health sector is “to fully exploit the beneficial effects of access to green areas” and park providers are to continue to provide adequate parks. 

Additionally, only a limited attempt has been made to provide a complementary economic assessment of parks. The economic value of parks in Australia is the value (expressed in monetary terms, i.e., in dollars) derived by Australians from the broad range of benefits generated by parks. The benefits of parks include health benefits that accrue to all Australians indirectly through the environmental benefits of parks such as the contribution to climate stability and water quality, but there are also important preventative and remedial direct health benefits gained by individuals who visit parks. Furthermore, even people who do not visit parks may still derive value from the option of using parks in the future or from the pleasure of the parks simply existing. This relative lack of evidence on the economic value of benefits to health and wellbeing is echoed in a (non-systematic) review of Australian and New Zealand evidence presented by Parks Forum, a peak body for parks organisations in Australia and New Zealand [20]. Examples of the results of valuation studies to date include over $7000 per hectare water purification benefit produced by permanent wetlands and over $100 million per annum water supply benefit to the Cotter catchment of Australian Capital Territory [21], a 20% premium on real estate values from location near a city park [22], and a contribution to local tourism and subsidisation of costs of staging community events of $130 million per annum for the Adelaide Parklands and over $10 million per annum across the Greater Sydney Region [23,24]. While this review demonstrates the size and scope of the potential total economic value of parks and open space in Australia, it also demonstrates the dearth of evidence on the value of health and wellbeing benefits from individual interactions. In light of this, the aim of this exploratory study was to estimate the perceived health and wellbeing benefits attained from visiting parks and the economic value assigned to parks by park users in Victoria, Australia. 

## 2. Materials and Methods

The research employed a mixed methods approach (park intercept survey and qualitative interviews) to collect primary data from a selection of park users. Data were collected between July 2015 and November 2015 on different days of the week and at various times of the day. Two researchers randomly approached English speaking adult park users inviting them to complete a short (10 min) written survey, resulting in 140 park user responses (80% response rate): 100 from two metropolitan Melbourne parks and 40 from a park on the urban fringe of Melbourne, Victoria. 

### 2.1. Brief Description of the Three Parks

Three parks were selected for inclusion in this study, as they represented a range of urban and peri-urban exemplars.

1. Park 1 is 55 hectares and consists of: natural bushland (less maintained than Park 2 and the park on the urban fringe), a playground (including old trams), BBQ facilities, public toilets, a homestead, a golf course, football ovals, and tennis courts, and has a main road as its entry point, a car park with a hard surface, and gravel paths throughout. This park is located in the eastern suburbs, approx. 19 km from Melbourne Central Business District (CBD).
2. Park 2 is 127 hectares and consists of: manicured lawns and natural bushland, a large lake, a café, three separate playgrounds, public toilets, BBQ facilities (rotunda), sporting facilities on site, asphalt paths (suitable for walkers and cyclists), and parking on site at the park (not asphalt). This park is located in the south-eastern suburbs, approx. 32 km from Melbourne CBD.
3. Park 3 is on the urban fringe of Melbourne and is 7.5 hectares and consists of: manicured lawns and natural areas (the most maintained), one playground, lake, BBQ facilities (rotunda), public toilets, a combination of asphalt and gravel paths, and parking on street (not as part of park). This park is located approx. 60 km from Melbourne CBD.

### 2.2. Surveys

The survey included measures of:

- the level and extent of the user’s engagement with the park;
- the attitudes and perceptions of park users about use and enjoyment of parks and the link to improved health outcomes;
- the importance of parks to users;
- the park user’s mental health (measured with the Perceived Stress Scale [25]) and wellbeing (measured with the Warwick-Edinburgh Mental Well-Being Scale [26]); and
- the economic value assigned by park users to parks.

A contingent valuation method was used to estimate the monetary value park users attach to the health benefits derived from parks. The survey used a dichotomous choice with follow up, in which participants were asked to imagine a hypothetical scenario where an annual levy is collected into a funding pool to maintain and provide access to parks in Victoria. At the same time, participants were reminded of the vital Fire Services Property Levy that is currently collected. Participants then made an economic choice whether they were willing to pay (WTP) (yes or no) with follow-up elicitation questions. A follow-up question to a response of “no” aimed to understand whether individuals were uninterested in the provision of parks or not (i.e., true zero values or protest responses). Another follow-up question to a response of “yes” invited individuals to decide on the maximum dollar amount they would be willing to pay for the provision and access to parks in Victoria. There were ten WTP interval bids ($0 to $100) to choose from, including an “other amount” which was an open-ended bid. 

Tobit regression [27] was used to examine the association between the WTP values and the participants’ demographic and socioeconomic characteristics. The Tobit model assumes that the dependent variable has a number of its values clustered at a limiting value, usually zero [28]. The advantage of Tobit regression is that it is able to estimate the relationship between the explanatory variable and some dependent variables (in this case WTP) to estimate the probability of a dependent variable being at or above a limit of $0. The explanatory variables are selected based on demographic factors found in the literature to influence WTP. Demographic factors, which could predict how WTP varies, were included in the model, such as age, gender, employment, and income [29,30]. We expected participants who frequented parks regularly, had children, and valued the provision of parks as highly important would exhibit higher WTP values than other respondents. Variables related to these attributes were also included in the Tobit model. Analysis was conducted in STATA/SE 14.0 (StataCorp LLC, College Station, TX, USA).

### 2.3. Interviews

Following completion of the survey, participants were invited to participate in a follow-up semi-structured one-on-one qualitative interview that lasted approximately 10–15 min and allowed them to expand on their responses to some of the survey questions. Participants were asked to describe, for example, how they felt about “access” to parks and the “benefits” parks may/may not provide them. The interviews were recorded and saved in accordance with ethical requirements and were transcribed verbatim. The interview data were thematically analysed, as informed by grounded theory [31]. The research team initially worked independently to code the data and establish themes and then came together to confirm and finalise the major themes and key findings, thus enhancing the rigor of the data analysis process [32]. 

### 2.4. Ethics

All participants gave their informed consent for inclusion before they participated in the study. The study was conducted in accordance with the Declaration of Helsinki, and the protocol was approved by the Deakin University Human Ethics Advisory Group approved the study (HEAG-H 69_2015).

## 3. Results and Discussion

The results of the study are presented in two parts: quantitative survey results and qualitative interview results. Where appropriate, the results are discussed in relation to previous research and ideas for future research are suggested. 

### 3.1. Quantitative Results

#### 3.1.1. Demographics

One hundred and forty individuals across three parks completed the survey. Selected demographic information of the study sample are presented in Table 1 below. The findings indicate that the majority of participants were female, aged 35–64 years, working full-time, with a weekly income of more than $1000 (AUD). They also tended to have children, own a dog, and have “very good” health. 

#### 3.1.2. Mental Wellbeing

One hundred and thirty-nine participants reported their mental health and wellbeing in the month prior to their park visit using the Perceived Stress Scale (PSS4) and the Warwick-Edinburgh Mental Well-Being Scale (SWEMWBS). The resulting score from the SWEMWBS ranged from 7 to 35 [33]. The range of the PSS4 score was 0 to 15 [34].

We did a statistical analysis of the mean differences between our means and the community sample/norms for an English sample, as no Australian data were available for comparison. The results were significantly different. The mean PSS-4 score reported by park users (M = 3.78, SD = 2.98) was significantly lower than the mean PSS-4 score (M = 6.11, SD = 3.14) reported by a general community sample in England [34], t(168) = 8.77, *p* < 0.001. The mean SWEMWBS reported (M = 19.78, SD = 2.75) was significantly lower than the mean score (M = 23.61, SD = 4.36) reported by a general population sample in England [35,36], t(145) = 16.27, *p* < 0.001.

Collectively, the results suggest that compared with the general population, park users experience lowered stress, yet rate their mental well-being as being poorer. In considering the potential inconsistences between the two outcome measures, the SWEMWBS captures a wider range of factors contributing to mental health over the previous two weeks, such as social relationships and view of the future, while PSS-4 primarily assesses stress over the last month. A possible explanation for the results may be that people who have recently been experiencing lowered mental well-being tend to go to the park to seek physical, mental, and spiritual benefits which may then lower their stress. The two constructs, however, may not be related.

#### 3.1.3. Park Visitation

A descriptive analysis of the participants’ use of parks are shown in Table 2. The findings indicate that 39% of park users visit parks to walk for exercise or to walk their dog (14%). They tend to visit parks with their partner (40%), children (25%), or with friends (27%). The findings also suggest that participants tend to visit parks for about 30–60 min, two to three times a week, to participate in light (51%) to moderate (36%) intensity physical activity, which is consistent with previous research (i.e., [7]). Additionally, they would “very much” miss the park if it was not around.

Park users’ valuation of parks and the future use of parks are summarized in Table 3. Eighty-four percent of respondents “strongly agreed” that having access to a park was important and 80% strongly agreed that in the future they might visit parks and their amenities. Most participants indicated (agreed/strongly agreed) that they used parks for physical activities (89%) and that visiting parks helped them to improve their feelings of wellness (98%). A high proportion also (agreed/strongly agreed) that parks provided an opportunity to value the environment as well as an opportunity to facilitate social interactions. These findings are consistent with previous research on the physical, social, and mental health benefits of park use [37]. 

#### 3.1.4. Willingness to Pay (WTP)

A total of 139 participants responded to the WTP valuation. A summary of the follow-up contingent valuation question is described in Table 4. Eighty-two percent of participants were willing to pay some annual dollar amount to keep parks. The highest monetary amount stated was $200 (AUD) per annum specified under “other amounts”. The most frequently reported monetary amount was $100 (AUD) per annum, was also the highest bid level offered (23%). Other popular responses were $20 (AUD) (13.7%) and $50 (AUD) (20.9%). These bid levels were equivalent to the three highest denominations of Australian banknotes (i.e., $20, $50, and $100). Including those not willing to pay, the overall mean (SD) annual amount park users were willing to pay was $45.4 (AUD) (38.4) to maintain and provide access to parks.

Table 5 represents the summary statistics of the mean WTP and standard deviation (SD) or the spread from the mean by the participants’ demographic characteristics and selected attributes related to attitudes and behaviour towards park use derived from the pilot survey. The variation in responses was fairly large, as seen in the standard deviation. Our mean WTP estimates identified men reporting higher mean (SD) WTP at $62.7 (AUD). Those employed stated higher WTP $52.0 (AUD) compared to unemployed $29.2 (AUD) and participants with higher income levels also stated the highest WTP $68.9 (AUD). Mean WTP amounts were also much higher in participants who visited parks daily $66.1 (AUD) and those who indicated vigorous activity levels during their park visits $67.8 (AUD). The mean WTP observed were also highest for those who visit parks for physical activities $49.3 (AUD), then social reasons $39.4 (AUD) and relaxation and wellbeing $23.3 (AUD). This is consistent with previous research that has found physical activity in the park to be of greater importance in predicting WTP than socio-economic or health predictors [29]. In addition, a majority of participants indicated that they would miss the park “very much” if it was not available, and their corresponding WTP amounts were also higher at $48.3 (AUD) than those who would “occasionally” or “never” miss it. In the same way, most participants considered the provision of parks to be “most important” or “just as important” as other local services, and were willing to pay higher amounts to keep parks.

The results from the Tobit regression are shown in Table 6. Demographic factors showed a mixed effect on WTP. As expected, low income was negatively related to WTP, and being employed was positively related to WTP. Being employed was associated with a 32.44 increase in the predicted value of WTP, however, these results were only marginally significant. Contrary to initial predictions, age showed a mixed effect on WTP. People aged between 25 and 65 years were less willing to pay to keep parks than people over 65 years of age. The reference category is the 65+ age category.

Furthermore, estimates from the Tobit model showed that the frequency of visits to the park and having children does not significantly influence park users’ WTP amounts. However, participants’ WTP as valued by provision of parks as “most important” or “less important” compared to other local services was significant at the 1% level. 

### 3.2. Qualitative Results

Seventeen participants agreed to be interviewed and five key themes emerged. The themes related to the health benefits derived from visiting parks and the factors influencing park visitation included: (1) health benefits, (2) access, (3) urban density, (4) children, and (5) safety. The following sections utilise participant quotes (denoted by P1–P17) to illustrate each of the key themes. The majority of the interview participants visited Metropolitan Park 1 (n = 11), followed by Metropolitan Park 2 (n = 4), and the park on the urban fringe (n = 2). They were predominantly of retirement age (n = 7) and female (n = 9). 

#### 3.2.1. Key Theme 1: Health benefits

This theme related to a range of health benefits that participants associated with visiting parks: physical, mental/spiritual, and social. Much like the literature on parks and health (refer to [37] and [9]), participants tended to associate their use of parks with various physical health benefits, some mental/spiritual benefits, and a few social health benefits.

- Physical

The majority of participants spoke of the physical health benefits derived from visiting parks in their neighbourhood. Many talked about the fact that they enjoyed walking in parks and being out in fresh air, as they felt that this helped their mobility (especially the older adult participants) and kept them fit. For example, P9 mentioned the benefits of walking in parks as follows:

“Walking is a great benefit for health, well we believe it is, and parks are a really nice place to walk.”

and P2 suggested:

“Well I think they help mobility, having just had a knee replacement two years ago and now I have just had a hip replacement, and I find walking regularly certainly helps with mobility, bring all my mobility back.”

Another participant, P3, also acknowledged the link between exercising in parks and physical health:

“One (benefit) is the health exercise, walking, the other is getting out in the fresh air and interacting with nature, birds, trees.”

Not surprisingly, and consistent with the literature [38], owning a dog was found to be a good conduit for walking in parks. Several participants acknowledged that they tended to visit their local park to exercise their pet. For instance, P7 commented:

“I come to walk the dog … when you’ve got a dog, especially a kelpie, dogs need exercise and you’ve just got to go out, whether you want to or not.”

Another participant, P13, commented that she spent time in parks to exercise her dog but also enjoyed the open space which was more preferable than exercising in an enclosed gym:

“Oh, just the exercise and the feeling of wellness you get when you’re out there in the fresh air… Mainly walking my dog… yeah just walks… it’s a nice place to exercise instead of standing in a gym, huffing and puffing and smelling all those sweaty people.”

Interestingly, some participants suggested that they also benefited mentally from time spent exercising in parks. For example, P1 stated:

“I’m moving for both physical movement sake but also for head space, head and heart space.”

and similarly, P5 commented:

“The physical benefits, like the exercise, is the benefit for everything, you walk, it starts from the bottom to head.”

Furthermore, P11 recognised the connections and suggested:

“The getting out, exercise, fresh air, getting around all that sort of thing… I think with a busy lifestyle visiting a park is a good thing to do exercise so I think exercise, for me, is quite important, you know, for my own sort of health but also, particularly, exercise is good for mental health as well.”

- Mental/spiritual

Whilst some participants identified mental health benefits as an additional benefit to their physical activity derived from exercising in parks, others indicated that mental health benefits were gained from just simply spending time in parks. For instance, P9 suggested:

“Even if you don’t go there to walk, if you just take a picnic and it’s just peaceful, helps everybody relax… We certainly do appreciate the trees and the bushland and the native flowers but I think that’s all part of the mental wellbeing thing, yeah.”

In congruence with the literature [37], most participants felt that parks provided them with a relaxing environment and a mechanism for stress relief. This was particularly important for those participants who lead busy lifestyles and work long hours. For example, P14 commented:

“I suppose it enables you to de-stress, put aside your own work or other stresses you might have in life so the benefit of going to a park is that a lot of those things are, not gone completely but are pushed back in your mind.”

Similarly, P8 eloquently highlighted the mental health benefits associated with visiting parks:

“I think just getting out in a park just refreshes people and gives people another perspective, whoever we are, if we’re just contained within four walls all of the time and we can get so desk-bound and so caught up with technology… Yeah, it gives you perspective and puts problems in perspective and yeah just helps just lifting depression a little bit too, anxiety, I think because less evil’s in our lives when we just get out into a park, for sure.”

Whilst not as commonly reported as mental health benefits, two participants recognised the associated spiritual health benefits resulting from their time spent in parks. P1 commented:

“Well, so I come from a faith background, so I’m a Christian, so I find being able to engage with the physical aspect of what I see around me takes away that sense of stuff in my head and just allows me to focus on God and creation and it’s just a pleasant place to be, just recharges my batteries.”

and P4 remarked:

“I think it’s just the peacefulness of it, yeah I love the peacefulness of it, you know I like the kids running around so it does, spiritually, if you’d like to say.”

The spiritual benefits derived from spending time in parks are generally not as well recognised as the mental (or physical) health benefits [37,39]. Therefore, these findings suggest this is an area that warrants further investigation.

- Social

Although not as commonly reported as physical or mental health benefits, social health benefits were identified by some of the participants. In particular, one participant who had recently migrated to Australia, indicated that they visited the park to meet people and another participant, P10, suggested that when they had a pet dog there were more likely to gain social health benefits:

“I used to go on my own a lot with the dog, we used to have a dog, and then I would make lots of new friends then, well new acquaintances.”

Another participant, P17, argued that parks are important for providing social health opportunities for families:

“Yeah definitely and to, ahh especially living in somewhat of a city area, being part of the community so maybe meeting people and like bringing kids and they meet people is good because I’m from the country where you know community, to be involved in the community seems to be easier in the country, you know, whereas here you’ve got to work a little bit harder at that and parks are good for that, yeah.”

On the other hand, several participants suggested that they did not associate spending time in parks with social health benefits. For example, P14 stated:

“I don’t go to the park to socialise as such, there may be benefits for others but not myself.”

Similarly, P1 remarked:

“I don’t think the social aspect of it really, when the children were smaller, for sure, we used to hang out at the playground and you’d meet other people… I don’t need to meet people but that’s not true for everyone, obviously.”

These findings are consistent with the literature [37,39] and indicate that social health benefits are generally not as well recognised as the mental (or physical) health benefits. Therefore, these findings suggest this is another area that warrants further investigation. However, we recognise that it would be difficult to quantify the social benefits in ways that may help parks managers justify retention and development of parks. 

#### 3.2.2. Key Theme 2: Access

This theme relates to the participants access to parks which was considered to be “very good” across Melbourne (they could visit a combination of larger parks and smaller neighbourhood pocket-parks). Predominantly, participants remarked that they lived within walking distance of a park and they highlighted how this proximity was beneficial. For example, P1 commented:

“I think we’ve got great access to parks in this area… having the ability just to be able to walk here is good.”

Similarly, P3 remarked:

“We have got this park close to home so that’s that is good and it’s a short drive to other parks.”

and P15 reported:

“Well, I think here I’m very lucky, compared to a lot of areas in the city, I’ve got quite large areas of parkland quite conveniently located nearby.”

Many of the participants suggested that proximity is critical to their park visitation, as commented by P14:

“In the case of Jells Park I can walk to it so proximity means it’s very accessible… Well I suppose proximity is the most important thing, if I had to drive somewhere I’d think twice about it, given I’ve got it right on my doorstep it’s not an issue.”

and P6 argued:

“They need to be within a reasonable distance too, sometimes you feel like going but you don’t want to spend half an hour getting there… I think it’s nice to have the availability to walk to a park, yeah, that’s probably important, a lot of families should have that, yeah, wherever they live.”

These findings are consistent with the literature which suggests that parks need to be within a short walk or drive from people’s homes in order for them to be regularly visited [8,39]. Furthermore, good access to parks is vital for people to reap the range of health benefits they can provide, especially the mental health benefits [39].

#### 3.2.3. Key Theme 3: Urban Density

This theme relates to the increasing urban density that many participants identified is encroaching on open space in their community. A number of participants, particularly those who lived near Metropolitan Park 1, identified the importance of available parks as housing density increases in their neighbourhood. For example, P1 commented:

“If we think that parks contribute to people’s mental and state of wellbeing then we should be investing in them and keeping these green spaces, especially as population and housing density, like increases along Burwood Highway and those other thoroughfares they need to keep parkland, people have to have somewhere to go, if they don’t have backyards, they’ve got to have somewhere to go.”

and similarly, P3 mentioned:

“I think as the city gets more densely built up parks are much more important for the fresh air, for the exercise, and for you know getting your feet on the ground.”

Other participants similarly highlighted the importance of parks in the inner-city environment, suggesting that they are vital to the health and wellbeing of people. For instance, P8 proposed:

“I just think they’re a really great asset that won’t always be there and the more we become urbanised the more we need green areas I think, they’re just so important for our mental health and wellbeing.”

and P7 commented:

“I just think they’re, you have to keep having parks, you can’t keep selling off the land and putting houses on it, you’ve got to have big green spaces, there’s got to be enough room for the trees to put more oxygen.”

These findings are consistent with the literature and emphasise that people strongly value the existence of parks in urban environments as they recognise the health and wellbeing benefits they provide [37]. This recognition is critical for planners and park managers to consider as part of managing open spaces and also for communities wanting to maintain their access to parks. 

#### 3.2.4. Key Theme 4: Children

This theme relates to “children”—in terms of them providing a reason for park visitation and in relation to whether parks adequately provide child friendly facilities. Some participants mentioned that they were more likely to visit their local parks to participate in physical exercise and play activities when they had young children. For example, P4 commented:

“When my daughter was young and we had sort of, you know, a few parties and all that sort of thing and they’d play on the swings or whatever, on the adventure playground, so I think it was probably more important then.”

and P1 mentioned:

“We used to come here a lot when our kids were small, now it’s often just me but on a good day, a few of us will come up, throw a Frisbee.”

Other participants spoke of the benefits parks provided their children, with a few respondents identifying the developmental benefits of children spending time in parks. For instance, P11 commented:

“It provides good engagement with children, that sort of park environment, it’s also quite good for their learning as well, you know learning how to do new things and explore their imagination so that’s probably quite a big and important thing for why I, or we, go to parks… interacting with other children but I suppose their development in terms of sometimes problem solving if the parks are difficult but all those, I suppose important things about, you know, that they get from going to a park, learning new things, engaging with different children, sharing, etc.”

Some participants discussed how they visited parks if they provided facilities that were appropriate for children such as, large areas for kite flying or ball throwing and safe bike tracks. One participant, P9, mentioned that he has found that as his children have grown:

“We’re finding they progress to different things in the park so it’s good that they have different things for a range of different children, you know, because you might have children and grandchildren of different ages go to the park, it’s no good if all of the stuff’s not suitable for one of them.”

Similarly, P10 commented that his use of parks has changed as he has acquired grandchildren:

“I’ve got the grandchildren here a lot, sometimes we’re using the parks to take them to so it’s just changing, my use of parks is changing I suppose…I’m changing the types of parks I’m using, we’re looking for more playgrounds than we had been, yeah… The type of equipment, I need it for the under-fives at the moment, but I, I’m also noticing that there are parks for older children that will come in handy later so it’s, it’s what I need at the time, are the ones for very small children.”

These findings suggest that the presence of children promotes park visitation, provided the accessible parks contain adequate facilities for children of varying ages. These findings support the literature that identifies a number of health, educational, and developmental benefits of nature contact for children [40] and the importance of suitable equipment for varying age groups [41,42,43,44].

#### 3.2.5. Key Theme 5: Safety

The final key theme derived from the qualitative findings was that of safety. The majority of participants felt safe in the parks they visited, but this was dependent on the time of day they visited, with some commenting that they would avoid visiting their nearby parks during the evening. For example, P13 mentioned:

“The only park that we don’t access, like later in the day, is the Gardiner’s Creek one because it’s not lit, it’s very dark and very eerie… anything could happen in those trees or bushes down there, you know it’s down, it’s in the middle of nowhere. During the day it’s all really safe.”

and P15 commented:

“I think that’s determined by when you go, I mean people do silly things… because I was coming back and it was dusk and I saw some people that I wasn’t too sure about so I went around but I mean that’s just being sensible, which everybody isn’t… I walked back through the park and it was pitch black and I felt a bit anxious, hmm, so I didn’t do that again.”

Some participants also discussed how they felt safe in their local park if other people were around them. For instance, P2 suggested:

“I think you just feel safer if you have got someone nearby you know. If you have a trip over you want someone there to pick you up.”

Similarly, P8 stated:

“I think people are never too far away in a park like this so safety’s pretty good, it’s got a pretty decent record, it’s a good area.”

These findings are consistent with the literature which indicates that visitation of parks is related to how safe people feel accessing them. For example, research by Koohsari et al. [45] found that factors such as perceptions of safety may influence the extent of walking in public open spaces and use by children and families [43,44].

## 4. Conclusions

This exploratory study represents an attempt to research the nature and extent to which parks can be publicly acknowledged as having very real and justifiable claims to being essential to health and wellbeing and that these claims can be justified in economic terms. A vital and central feature of the study is that it focused on people who use parks to gain some assessable insight into their views on the need for, and economic value they place on, parks. Our findings suggest that park users derive a range of physical, mental/spiritual, and social health benefits. Park use was predominantly associated with physical health benefits, followed by social benefits and mental health benefits. Participants were found to “highly value” parks to improve mood and feelings of wellness. 

In terms of the participants’ willingness to pay for the maintenance of and access to parks, our findings suggested that the overall mean (SD) annual amount park users were willing to pay was $45.4 (AUD) (38.4). Predominantly, participants considered the provision of parks to be “most important” or “just as important” as other local services and were willing to pay higher amounts to keep parks. 

### Limitations

Some caution needs to be taken when interpreting our findings, as they are from a relatively small sample of parks users and therefore the results could be somewhat biased towards those who visit parks. Our results are also limited by the fact that data was only collected from three parks which offered a diverse range of facilities and attributes (i.e., sporting facilities and playgrounds), so it is possible that they provided more opportunities for physical activity than parks in other neighbourhoods. In addition, our sample did not include participants from different cultural groups or people from various socio-economic statuses, so our results are not representative of the general population. Furthermore, given the lack of published Australian norms and the meaning of differences in the mental health score, the magnitude of the scale scores on PSS4 and SWEMWBS should be interpreted with caution. We also acknowledge that due to only 17 participants being interviewed, we are not able extrapolate our qualitative findings to the general population.

However, overall our exploratory study findings suggest that park users are willing to pay for parks, as they highly value them as places for exercising, socialising, and relaxing. Importantly, most people would miss parks if they did not exist. The findings aim to provide park managers, public health advocates, and urban policy makers with evidence about the perceived health and wellbeing benefits of park usage and the economic value of parks. 

## Figures and Tables

**Table 1:** Background characteristics of survey sample.

Baseline Characteristics	Summary	
Survey sample, n	140	
Male, n (%)	41	(29%)
Age group, n (%)		
18–34 years	32	(23%)
35–64 years	74	(53%)
65+ years	33	(24%)
Studying, n (%)	21	(15%)
Employment status, n (%)		
Full-time employed	41	(29%)
Part-time employed	35	(25%)
Unpaid work	14	(10%)
Unemployed—seeking work	4	(3%)
Unemployed—not seeking work	6	(4%)
Retired	38	(27%)
Average size of household, (SD)	2.9	(1.2)
Dog ownership, n (%)	50	(36%)
Have children, n (%)	108	(77%)
Have grandchildren, n (%)	32	(23%)
Household income range (in AUD) per week, n (%)		
<$1000	21	(15%)
$1000–$1999	35	(25%)
$2000+	28	(20%)
General health		
Excellent	35	(25%)
Very good	68	(49%)
Good	32	(23%)
Fair	4	(3%)

**Table 2:** Description of park visitation.

	n	%
Use of parks and facilities	130	93
Main reason for park visit		
Walk	54	39
Walk the dog	20	14
Ride a bike	2	1
Jog/run	7	5
Ball games	2	1
Other exercise	0	0
Supervise children	10	7
Spend time with family/friends	13	9
Picnic/BBQ	16	11
Socialise	1	1
Attend event/celebration	2	1
Visit café	2	1
View nature	2	1
Relax	0	0
Other	8	6
Accompaniment to park		
Alone	28	20
Partner or other family members	56	40
Child(ren)	35	25
Grandchild(ren)	8	6
Friends	38	27
Organised group	15	11
Dog	18	13
Other	2	1
Time spent in park on day of survey		
<30 min	16	11
30 min–1 h	58	41
1–2 h	53	38
2–3 h	11	8
3–4 h	2	1
4+ h	0	0
Average number of park visits in the past 3 months		
Daily	9	6
Two to three times/week	28	20
once/week	19	14
Two to three times/month	22	16
Once/month	15	11
<Once/month	12	9
First time to the park	34	24
Other	1	1
Usual physical activity levels in this park in the past 3 months		
Mostly sitting	10	7
Mostly light activities	71	51
Mostly moderate activities	50	36
Mostly vigorous activities	9	6
Would you miss this park if it was not around?		
Very much	107	76
Occasionally miss it	29	21
Never, wouldn’t notice if it was not there	2	1

**Table 3:** Value of parks.

Value of Parks and Future Park Use (%)	Strongly Agree	Agree	Neither	Disagree	Strongly Disagree	Do Not Know
Having access to park is important to me	84	14	1	1	0	0
Having access to park is not important to me	0	0	1	22	75	0
In future, I might visit/use parks and their amenities	80	18	0	0	1	0
In future, parks will become important to me	71	21	2	0	1	4
I use parks for physical activities	68	21	1	7	1	0
Parks provide an opportunity to learn/value environment	36	43	11	6	1	1
Parks provide an opportunity for social interactions	21	44	16	15	1	1
Parks help improve mood	58	40	1	0	0	0
Visiting parks help improve feelings of wellness	64	34	2	0	0	0

**Table 4:** Reported annual willingness to pay (WTP) for the provision and access to parks in Victoria, Australia. All dollar values listed are in AUD.

WTP Bid	n	%
$ amount	25	18.0
$5	5	3.6
$10	5	3.6
$15	6	4.3
$20	19	13.7
$25	1	0.7
$30	9	6.5
$50	29	20.9
$70	7	5.0
$100	32	23.0
$200	1	0.7
Total	139	100.0

**Table 5:** Park users WTP by demographic characteristics and survey responses. All dollar values listed are in AUD.

	WTP		
	n	Mean	SD
Gender			
Male	41	$62.68	$38.72
Female	98	$38.11	$36.05
Age			
18–24	15	$56.67	$39.22
25–34	17	$25.00	$27.89
35–44	23	$41.30	$32.48
45–54	25	$59.60	$46.68
55–64	26	$47.69	$41.98
65+	33	$40.91	$32.94
Pet dog	50	$48.70	$42.56
No dog	88	$42.84	$35.66
Employment			
Employed	76	$52.04	$40.82
Unpaid work, unemployed	24	$29.17	$29.73
Retired	38	$40.79	$34.77
Income			
<$1000 per week	21	$37.86	$32.31
$1000–$1999 per week	35	$42.71	$35.22
$2000+ per week	28	$60.89	$39.21
Children			
No children	31	$41.13	$36.67
Children	108	$46.57	$38.96
No grandchildren	89	$45.90	$39.27
Grandchildren	32	$39.84	$36.02
General health			
Excellent	35	$37.71	$38.35
Very good	68	$41.69	$33.94
Good	32	$60.47	$42.91
Fair	4	$53.75	$53.75
Main reason for park visit			
Physical activity (e.g., walk, jog, cycling, games)	85	$49.29	$40.70
Social (e.g., picnic/BBQ, visit café, event/celebration)	45	$39.44	$34.03
Emotional-wellbeing	3	$23.33	$25.17
Other	5	$54.00	$36.47
Frequency of park visits in the past 3 months			
Daily	9	$66.11	$35.34
Weekly (one to three times/week)	47	$38.30	$34.83
Monthly (one to three times/month)	37	$50.54	$36.57
Less than once/month	12	$48.75	$42.22
First time to this park	34	$42.79	$43.47
Usual activity level in the park in the past 3 months			
Mostly sitting	10	$39.00	$38.14
Mostly light	70	$42.29	$34.89
Mostly moderate	50	$46.90	$43.18
Mostly vigorous	9	$67.78	$34.20
Miss park if not around			
Very much	107	$48.27	$39.36
Occasionally	28	$35.71	$35.71
Never	2	$35.00	$21.21
Park importance compared to other services			
Most important	11	$37.27	$37.97
Just as important	113	$47.57	$39.22
Less important	12	$28.33	$28.23
Not sure	3	$60.00	$36.06

**Table 6:** Tobit regression results.

Variables	Coefficient	Standard Error	*p*-Value
Gender			
Female	−4.29	8.93	0.63
Age			
18–24	0.07	20.91	1.00
25–34	−57.41	20.09	0.01
35–44	−31.99	20.03	0.12
45–54	−41.42	21.41	0.06
55–64	−35.85	17.49	0.04
Employment			
Employed	32.44	18.32	0.08
Unpaid work, unemployed	7.27	18.46	0.70
Income			
<$1000 per week	−23.20	12.58	0.07
$1000–$1999 per week	−11.86	9.79	0.23
Children	16.32	11.50	0.16
How often visited park in the past 3 months			
Daily	46.33	35.73	0.20
Weekly (one to three times/week)	16.73	10.54	0.12
Monthly (one to three times/month)	3.77	11.85	0.75
<Once/month	14.69	14.25	0.31
Park importance to other services			
Most important	−83.13	32.95	0.01
Just as important	−30.83	27.23	0.26
Less important	−83.32	30.81	0.01
Constant	81.43	37.44	0.03
n	83		
Pseudo-R2	0.051		
Log likelihood	–352.7		
Prob > chi2	0.004		
Mean WTP	$43.47		
95% CI	$36.14	$50.80	
